# Complications of Gonorrhea and Treatment*Read before meeting of the East Texas Medico-Chirurgical Association, May, 1906.

**Published:** 1906-11

**Authors:** E. B. Parsons

**Affiliations:** Palestine, Texas


For Texas Medical Journal.

### Complications of Gonorrhea and Treatment.*

BY E. B. PARSONS, M. D., PALESTINE, TEXAS.

In glancing over the anatomy of the penis, we can readily see why we are prone to have complications resulting from an inflammation of that organ, and more so if specific in origin, for the urethral canal is pierced at all points by ducts leading from some special gland having a specific function to perform; hence the introduction of the gonococci into these various channels is quite the natural thing to suspect and in the majority of cases does occur.

The first we may encounter in this disease is balanitis posthitis. These are so intimately connected that they may be taken up and discussed as Balano-posthitis, which is inflammation of the gland

*Read before meeting of the East Texas Medico-Chirurgical Association, May, 1906.

and mucous surface of the prepuce, and it is usually caused by neglect in cleanliness, but sometimes as a direct extension regardless of care, and especially those of rheumatic and gouty tendency. The symptoms, briefly stated, are heat, redness, swelling, accompanied by a burning and itching sensation and exfoliation of epithelium, and excoriation, leaving irregular *ulcers; these ulcers must not be mistaken* for *chancroid.*

*Treatment.*—Soap and water; bichlorid solution and some good dusting powder; avoid friction.

*Phimosis.*—An inflammation of the prepuce is usually associated with Balano-posthitis, and is frequently an objectional complication. The symptoms are practically the same as above, save swelling, which may extend to abdomen.

*Treatment.*—Elevation of penis and wet dressing; retraction of prepuce and clean out cavity. If the above treatment fails, circumcise.

Para phimosis is a more dangerous complication, and does not occur quite so often; the return of the blood is obstructed from the glans-penis, causing swelling, ulceration and gangrene. The first steps in the treatment is by warm application; if this does not reduce the inflammation, apply forcible reduction; this method is simple and effective, and will not be referred to here, but if the ordinary methods fail, surgical interference must be resorted to, such as cutting off the edematous tissue in front of constriction and applying ice or a complete severance with a tenotome.

*Follicular and peri-urethral abscess* is an inflammation of the follicles and surrounding tissues which, in the course of gonorrhea, results from occlusion of the mouth of the many follicles along the urethral canal. This is usually brought about by aggravation of the existing gonorrhea from excessive drinking, frequent erections, too strong injections, and instruments. This we very often encounter, and it has its important bearing upon the treatment in its first stages, and should be noted carefully; if excessive, it may result in what is termed peri-urethral abscess. They may discharge pus in the urethral canal or may open externally; if not, should be incised and drained, but resolution is the usual mode of termination.

Lymphangitis is sometimes a complication and may present itself in two varieties, viz.: an enlarged lymphatic chord with absence of ordinary symptoms of inflammation; and again there will be all the signs of inflammation accompanied by edema of prepuce and enlargement of the inguinal glands.

*Treatment.*—Wet dressings, hot baths and application of ichthyol. All these may be the advance guard of what is called gonorrhea bubo, which is an enlargement or enlarged suppurating inguinal gland. The treatments depend upon the condition of the bubo; when the gland is slightly enlarged and moves freely under the skin, treatment is unnecessary, but infiltration of the tissues results, and suppuration makes its appearance. Incision is the treatment.

*Cowperitis.*—This is an inflammation of Cowper's glands, situated in the perineum, close to the triangular ligament, and usually become inflamed about the fourth week as a result of frequent catheterism, sexual intercourse, and other irritations. The diagnosis of this condition is made by palpating the perineum, which will reveal a small lump tender to touch on one or the other side of the median line. It may disappear under treatment, but sometimes goes on to suppuration, which intensifies the symptoms, causing great pain, swelling, difficult and painful urination, and painful walking. If suppuration does occur, it may end by discharging through the urethra or point to the skin surface and be discharged.

The differential diagnosis between Cowperitis and urinary perinéal abscess must be made. Urinary abscess usually follows stricture of urethra or external violence to the penus, and is slow in onset and is always central, while Cowperitis is always upon one side of the median line; these must be carefully noted in order that a proper diagnosis can be made.

The treatment consists in rest, hot baths, laxatives, local applications, poultices, ichthyol ointment, as stages seem to indicate. If suppuration occurs, make a very free incision; owing to the peculiar anatomy of the gland, you may not reach all the pockets of pus, hence an end incision should be made.

Prostatitis, one of the most important and a very frequent complication of gonorrhea, occurs during the course of posterior urethritis. From the passage of instruments, sexual excitement, alcoholic excess, exposures, or from simple extension of inflammation, the condition is usually ushered by a chill, followed by sense of heat, distension, weight in the perineum and rectum, and is followed by frequent urination, burning urine, both before and after the act of defecation, and is also painful. Sometimes fever and chill is very intense. If you will examine the rectum you will reveal a large mass and tender to the touch; as the disease progresses the pain increases and becomes throbbing, and will radiate to the neighboring regions. Defecation is difficult and painful, also urination is frequent, and may be complete retention. The condition may end in resolution, leaving the prostate chronically enlarged, or may suppurate.

These conditions, gentlemen, I think, are responsible in many instances for the prostatic troubles in old men. Another important diagnostic feature is, when pus is present it may discharge through the urethra and *only* be present during the urinary act, as the contraction of the cut-off muscles prevents the escape of pus during the interval; it may point through the rectum or perineum.

The treatment of prostatitis to abate the inflammation by rest, avoids the passage of hard instruments; in case of retention, use gently a soft rubber catheter and anchor; this is better than frequent use of hot water douches in rectum and fomentation to perineum; *never* use massage in acute cases; anodynes if pain is severe.

Spermato cystitis is an inflammation of the seminal vesicles and usually accompanies prostatitis, posterior urethritis, and epididymitis. The symptoms accompanying the acute variety are usually overshadowed by the complications. It is usually associated with and passes unrecognized, but severe cases may be recognized by fever, rectal touch, local pains in perineum, anus and hips, per rectum, and will be found above the prostate and to one side, a hot, soft, fluctuating, sausage-like mass, pains radiating to testicle, neuralgic pains along the prostatic sinus, irritability of the sexual organs, desire increased and ability diminished. It is in this complication that we find most of our sexual neurasthenics, the condition becoming chronic, atrophy of the vas and epididymis, and we have a hopeless picture of the sexual hypochondriac to deal with.

The treatment of acute vesiculitis is absolute rest in bed, opium suppositories (blood in the semen), camphor monobromid to modify erection, local application of heat, warm enemata, and after-effects combated by general sexual hygiene and tonics. *All* examinations per rectum should be avoided.

The chronic condition is best treated by hot water douche, and if they fail, massage carefully and preferably with the index and middle fingers once every five days.

Epididymitis and orchitis usually are associated and produced by extension of inflammation caused by patient lifting a heavy weight, a horseback ride, and unusual venereal excitement, and occurs during the fifth and sixth weeks; it usually renders the patient bed-ridden and is so prone to relapses that the most trivial causes may effectually light up all the symptoms again and again. It is usually unilateral, but may extend to its fellow without provocation, hence the treatment must of necessity be a confining one; rest in bed flat on back, lead and opium lotions, strapping the testicles up to relieve the chords of weight, poultice of tobacco, ichthyol, mercury and iodin, unguents locally applied; pain must be combated by anodynes, of which morphin ranks first. After the acute symptoms have subsided a more stimulating application is indicated,—strapping the testicles is recommended by some, but I find it is of no more use than the remedies mentioned above. All urethral medication is contraindicated during the acute attack, but constitutional treatment must be instituted. The chronic inflammation of the globus minor frequently causes sterility.

Gonorrheal cystitis, a frequent complication of posterior urethritis, occurs during the third week and after, and usually involves only the vesical neck. The main symptoms of recognition are a drop or two of blood at the end of micturition, a constant desire to empty the bladder, associated with burning pain instead of relief. Blood and pus may follow the urine and always turbid.

*Treatment.*—Rest, alkalin diuretics,—stop all urethral treatment, —opium suppository, local heat. It is very difficult to tell just when to institute local treatment again, but if the symptoms above enumerated are not remedied I would begin permanganate pot. solution, 1 to 4000, thorough irrigation, or 20 gtts. 1 to 500 solution nitrate of silver internally.

Finally, gentleman, last, but not least, we have a general septicemia infection, caused by the gonococci, a systemic infection called gonorrheal rheumatism, of which the pathology is somewhat in doubt, but usually manifests itself in people who are of gouty or rheumatic tendency, and a complication of septic endocarditis. For the symptoms and treatment I will omit, as time will not permit of discussion here.

